# Autologous platelet-rich fibrin (PRF) augmentation as an add-on therapy in deep surgical site infections (dSSIs) after instrumented spinal surgery: preliminary results of a single institution case series

**DOI:** 10.1007/s00701-021-04952-7

**Published:** 2021-08-24

**Authors:** Ioannis Vasilikos, Roland Roelz, Christoph Scholz, Boris Mizaikoff, Katerina Argiti, Watzlawick Ralf, Georgios-Christos Giagkos, Evangelos M. Fragkakis, Shahram Ghanaati, Jürgen Beck, Ulrich Hubbe

**Affiliations:** 1 Department of Neurosurgery, Medical Center, University of Freiburg, Freiburg, Germany; 2 Institute of Analytical and Bioanalytical Chemistry (IABC), University of Ulm, Ulm, Germany; 3 School of Medicine, National and Kapodistrian University of Athens, Athens, Greece; 4 Department of Neurosurgery, Medical Center, St George’s University Hospital, London, UK; 5 Department of Maxillofacial and Plastic Surgery, Medical Center, University of Frankfurt, Frankfurt, Germany

**Keywords:** Surgical site infection, Spinal surgery, Platelet-rich fibrin, Autologous biomaterial

## Abstract

**Background:**

Deep surgical site infections (dSSIs) after instrumented spinal surgery pose major therapeutic challenges. Standard treatment involves surgical debridement, wound drainage, and long-term antibiotic administration. Autologous platelet-rich fibrin (PRF) constitutes a biomaterial obtained from patients’ own blood that contains leukocytes, chemokines and growth factors boosting cicatrization. Due to favorable results reported from other surgical disciplines such as dentistry, orthopedics, maxillofacial and plastic surgery using PRF, the authors hypothesized that PRF augmentation will promote wound healing in dSSIs.

**Objective:**

To report our preliminary results on the safety and efficacy of autologous-PRF as an add-on therapy on a pilot case series of persistent dSSI after instrumented spinal surgery.

**Methods:**

Among the 293 patients who underwent dorsal decompression and stabilization of the cervical, thoracic, and lumbar spine due to degenerative diseases in our department, 12 patients (4%) presented persisting dSSI after standard wound debridement and antibiotic treatment. PRF augmentation was used during a second surgical revision as an add-on therapy to standard debridement. In all cases, the wound was primarily closed without drains.

**Results:**

Wound healing was completed between 14 and 21 days after the second surgical revision in all patients. At a median follow-up of 8 months (range: 6 to 18 months), no recurrence of dSSI nor complications were encountered in any case.

**Conclusions:**

Our preliminary results suggest that PRF augmentation in persistent dSSI after instrumented spinal surgery appears to be a safe and effective strategy to promote wound healing. Prospective controlled studies are required to define the efficiency of PRF more clearly in both treating and preventing dSSI.

## Introduction

Surgical wound healing is a complex process influenced by both exogenous and endogenous factors such as: length of the incision, surgical duration, pathogens’ colonization with pathogens, diabetes, smoking, local biology/vascularization (e.g. revision surgery, skin affected by radiotherapy) and nutritional status [7]. With an incidence of up to 12%, post-operative spinal infections constitute a frequent complication [4, 6]. The Center for Disease Control and Prevention classifies the surgical site infections (SSIs) after spinal surgery into superficial and deep, located epifascially (skin and subcutaneous tissue), and subfascially (epidural abscess, spondylitis, spondylodiscitis), respectively [11, 32]. Deep SSIs is particularly observed after extensive exposures and metalwork implantation, often leading to soft tissue defects, impairing patients’ recovery with significant socio-economic costs [4, 6].

**Fig. 1:**
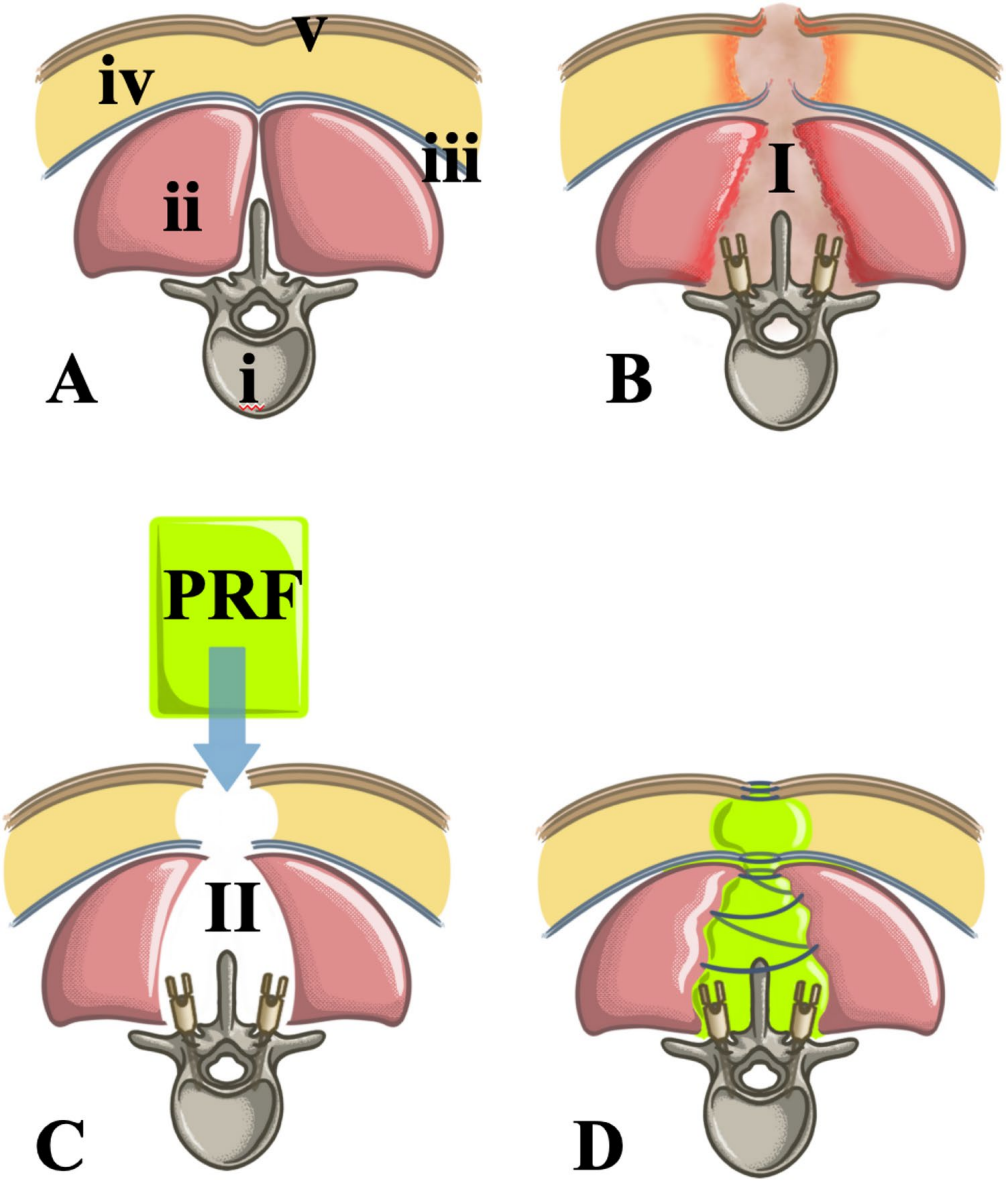
**A** Axial section of a healthy lumbar spine, i, Vertebral body; ii, paravertebral structures; iii, Fascia; iv, subcutaneous fat; v, dermis and subcutis. **B-I** Deep surgical site infection with tissue destruction. Left and right to the spinal process are represented the implanted posterior instrumentation. **C** Axial view after extensive debridement and resection of infected and necrotic tissues. The residual tissue defect is C-II. The autologous platelet-rich fibrin matrix is green. **D** Platelet rich fibrin augmented tissue defect after re-adaptation of the paravertebral structures and primary wound closure

There are no well standardized management protocols for the treatment of dSSIs. Most widespread practices include extended wound debridement, closed-suction drains and identification of pathogens with subsequent targeted antibiotic treatment [6, 24, 31]. However, reconstruction of paravertebral soft tissue and wound healing may be challenging after extensive debridement, since necrotized space fills with a mixture of fluid and wound debris (Fig. 1,1B). This environment noticeably impairs cell-migration and proliferation leading to persistent dSSI, particularly in the presence of comorbidities interfering with the physiological wound healing [28]. Since primary wound closure frequently is not possible for these reasons, antimicrobial moisture-retentive dressings and vacuum therapies are commonly applied [28].

Platelet-rich fibrin (PRF) constitutes an autologous biomaterial obtained from the patients’ own peripheral blood after centrifugation. Consisting of a fibrin matrix containing high concentrations of platelet-derived growth factors, chemokines, and leukocytes were able to enhance wound healing and to provide local antimicrobial protection [13, 16, 17, 29]. Due to these biological properties, PRF was used in restorative dentistry, orthopedics, maxillofacial and plastic surgery, as well as for the treatment of persistent diabetic ulcerations with promising results [2, 5, 33, 14, 22, 30]. Therefore, we hypothesized that PRF may be an appropriate biomaterial to promote wound healing in deep spinal infections which failed to respond to the traditional surgical approach (wound debridement, drains, antibiotics), especially in the presence of spinal metalwork. Favorable results observed in our consecutive case series, encouraged us to report on our findings.

## Patients and methods

### Patients

During a two-year period in our department (12/2017–11/2019), a total of 293 patients underwent dorsal decompression and stabilization of the cervical, thoracic, and lumbar spine due to degenerative pathology. Surgical site infections (SSIs) occurred in 24 patients (8%), of which 4 (1%) were superficial and 20 (7%) were deep infections. While superficial infections resolved after conservative treatment with antibiotics in every case, all patients with deep surgical site infections (dSSIs) underwent extended surgical debridement (first surgical revision) and intravenous (IV) pathogen-specific antibiotic therapy. Although in 8/20 cases, dSSI resolved with this therapeutic strategy, 12/20 patients (4%) failed to respond with persistent purulent secretion and wound dehiscence, thus were included in our study. After another debridement (second revision) and continuous antibiotic therapy, PRF augmentation was performed as an add-on therapy in these 12 patients. Details on surgical procedures and PRF augmentation are given in the respective paragraphs.

### Initial surgery

Of the 12 patients, 6 (50%) were operated in the cervical, 5 (42%) in the lumbar and 1 (8%) in the thoracic spine via a dorsal approach, which was minimally-invasive in 4 cases (33%). Titanium implants were used across the cohort. According to our local policy, patients received as prophylaxis during the anesthetic induction 2 g Cefuroxime or alternatively 500 mg Clindamycin, if allergic to penicillin and if needed repeated intraoperatively every 4 h and 8 h, respectively. The surgical site was cleaned with Betadine or Chlorhexidine scrub. During revision, dural tear was identified in 5 patients (41.7%). Surgical drains were placed and removed when the drainage was less than 50 ml/day.

### First surgical revision

Deep SSI was confirmed by contrast-enhanced MRI and laboratory tests including blood cell counts, erythrocyte sedimentation rate and C-reactive protein. The germ spectrum was determined by wound swabs and blood cultures. Extensive wound washout with Ringer´s solution and meticulous surgical debridement of all devitalized tissue was performed in every case. Wound was primarily closed in layers, leaving in situ two drains. In all cases the metalwork was preserved and a pathogen-specific antibiotic therapy was performed.

During the 4 weeks follow-up period, this standard approach failed to resolve the dSSI in 12 cases as confirmed by clinical laboratory and radiological assessments. Therefore, PRF augmentation was planned as an add-on therapy during the second debridement after acquiring patients’ informed consent.

### Preparation of platelet-rich fibrin (PRF) as solid (s-PRF) and injectable (i-PRF) biomaterial

For the preparation of PRF, 120 ml of patient’s own peripheral blood was collected intraoperatively using a standard butterfly. The blood was centrifuged in a DUO centrifuge at 1200 rpm for 8 min (177 g) using a fixed angled rotor with a 110 mm radius (Process for PRF, Nice, France) according to the low centrifugation concept protocol of Choukroun and Ghanaati [13]. This protocol ensures a high concentration and homogenous distribution of autologous platelet-derived growth factors in PRF [13]. For every wound augmentation, 6 s-PRF vials and 6 i-PRF vials were used, each containing 10 ml of donor-matched blood in glass and plastic tubes, respectively (Process for PRF, Nice, France). After the completion of the centrifugation cycle, the tubes were left in the device to settle for 5 min.

### Second surgical revision

**Fig. 2:**
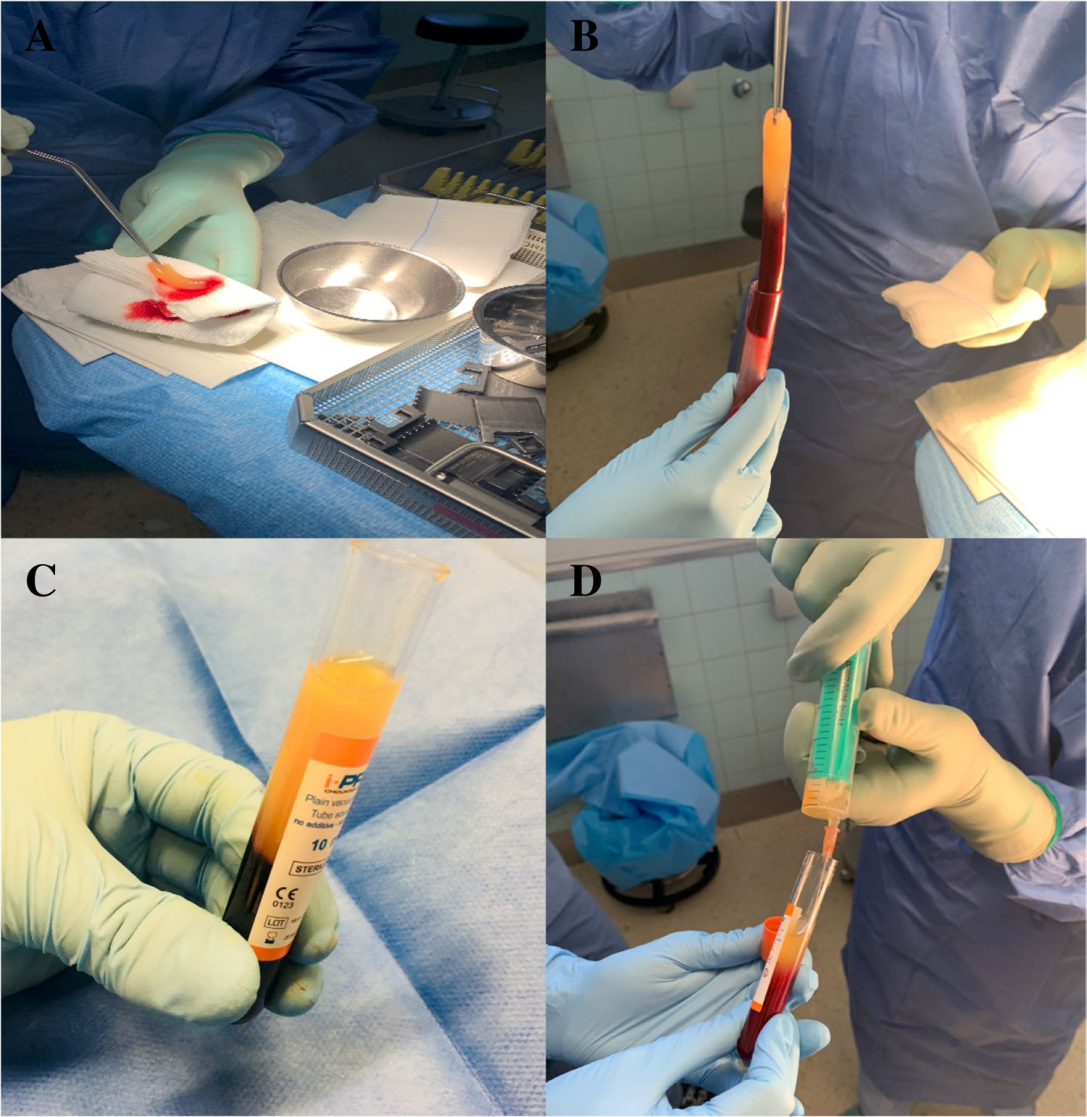
**A** Autologous solid platelet-rich fibrin matrix. **B** Collection of the solid platelet-rich fibrin matrix from its glass vial after centrifugation. **C** Injectable platelet-rich fibrin after centrifugation in its plastic vial. **D** Collection of the injectable platelet-rich fibrin in a 20-ml syringe

**Fig. 3:**
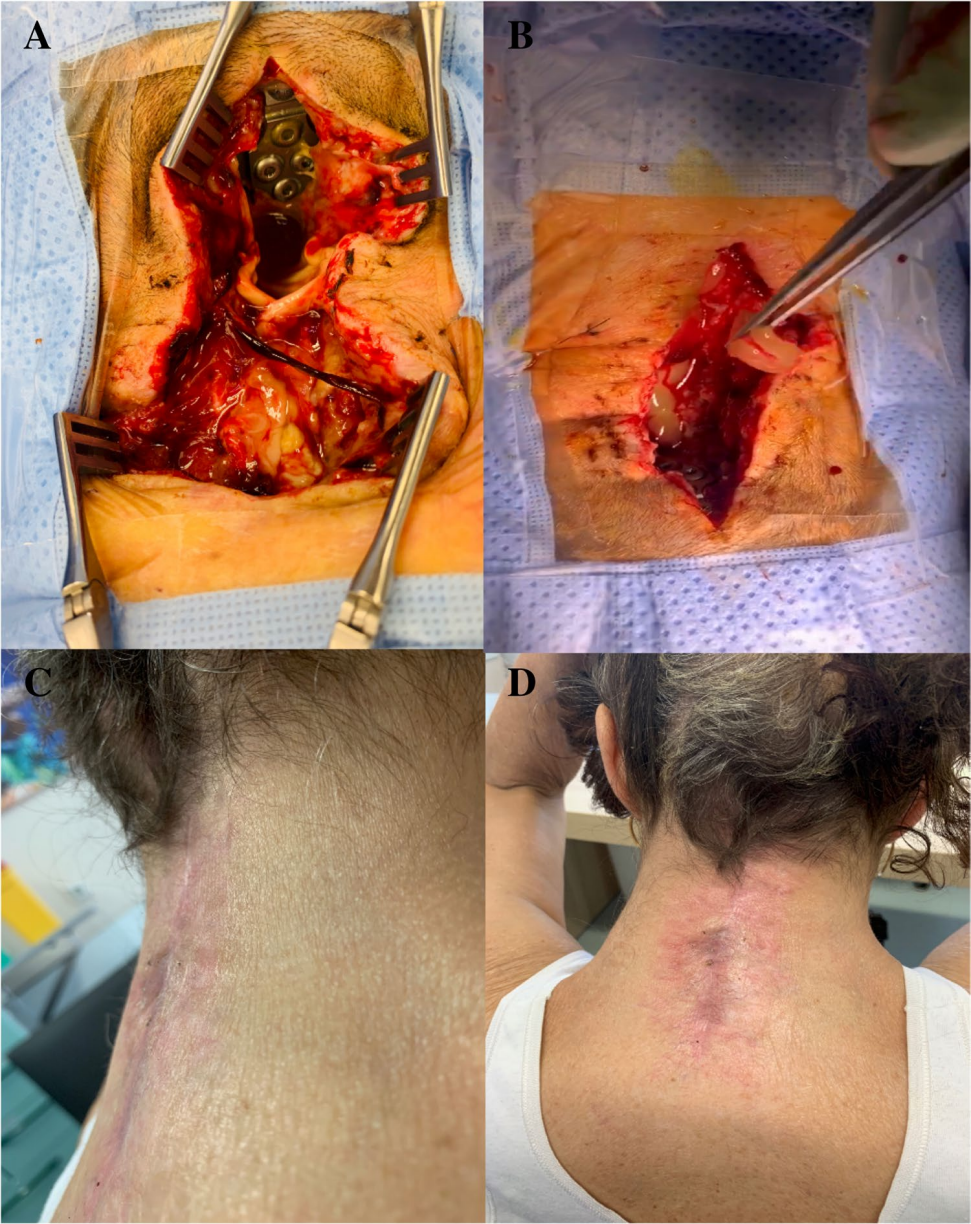
Representative case from our study group. Craniocervical posterior fixation was performed. **A** Intraoperative posterior view of the deep surgical site infection. The instrumentation is visible. **B** After extensive debridement, the residual tissue defect was augmented with solid and injectable platelet-rich fibrin. Primary wound closure was performed without closed suction drains. **C** and **D** Lateral and anteroposterior view of the cervical spine 19 days after platelet-rich fibrin augmentation

After renewed debridement, the residual tissue defect was augmented with autologous PRF in both solid and injectable forms (Fig. 1, 1C, 1D). From the glass tubes, s-PRF was collected (Fig. 2, 2A, 2B) and inserted in the tissue-defect (Fig. 3, 3B). From the plastic tubes, the thick fluid injectable PRF (i-PRF) was aspired in a 20-ml syringe and filled the remaining tissue-defect (Fig. 2, 2C, 2D).

Revision of spinal instrumentation was not required in any case and drains or local antibiotics were not used. Primary wound closure was performed in every patient. The pathogen-specific IV antibiotic regimen was continued. After the normalization of the C-reactive protein (CRP) values, the IV antibiotics were switched to oral continuing for 2 more weeks.

Subsequent follow-ups for wound checks and laboratory tests were performed twice a month for 3 months, including clinical, neurological assessments and CT/MR imaging, as needed. All patients were scheduled to have CT-scans 12 months postoperatively to evaluate bone fusion. Observation time ranged from 6 to 18 months (median: 8 months).

## Results

There were 7 females and 5 males with a mean age of 66.6 ± 5 year. Obesity defined as a body mass index (BMI) > 30 was observed in 7 (58.3%) cases. Four patients (33.3%) had diabetes.

Staphylococcus aureus was identified as the leading pathogen by swabs and blood cultures in all 12 patients at the first surgical revision and all of them received tailored antibiotic therapy.

The postoperative course after the second surgical revision with PRF augmentation was uneventful in every patient and completed wound healing was achieved between 14 and 21 days in all cases. Postoperative in-hospital stay was 18.3 ± 2.7 days. No recurrent SSI and no complications were noted at a median follow-up of 8 months (range: 6–18 months) in any case. Postoperative CT-scans at 12 months revealed distinct signs of bony fusion in all cases. Loosening of single screw was noted in 2 patients, however no surgical intervention was required, since they were virtually asymptomatic and presenting sings of advanced bony fusion on CT scans. At last follow-up, all patients presented satisfying outcome without additional neurological deficits and normal inflammation parameters.

## Discussion

Instrumented spinal surgery is a commonly utilized procedure, which however is not devoid of complications, especially when it comes to patients with associated comorbidities or elderly, which could lead to surgical site infections (SSIs) [8]. Foremost, diabetes-associated systemic microvascular impairment can reduce the local ability to resist infection [19], and leukocyte functions (adherence, chemotaxis and phagocytosis) are undermined by hyperglycemia [9]. Furthermore, diabetes can disturb the collagen synthesis and fibroblast proliferation, thus delaying the wound healing process [3]. Nicotine induces peripheral vasoconstriction and tissue hypoxia resulting in impaired local angiogenesis and epithelialization [23]. Obesity, surgical invasiveness, and implantation of foreign material have been identified as independent risk factors for deep SSIs (dSSIs) [15]. Thus, dSSIs constitute a major problem in instrumented spinal surgery, especially in elderly patients when larger exposures are required.

Favorable results in other disciplines such as dentistry, orthopedics, plastic and maxillofacial surgery, as well as in the treatment of persistent diabetic ulcerations [2, 5, 33, 14, 22, 30] encouraged us to focus on the use of biomaterials with high concentrations of autologous platelet-derived growth factors that can augment the residual tissue defect and provide an active cell-migration matrix. Platelet-derived growth factors such as vascular endothelial growth factor (VEGF), keratinocyte growth factor (KGF), insulin-like growth factor (IGF), epidermal growth factor (EGF) and transforming growth factor-β (TGF-β) are found at high concentrations and homogeneously distributed in the fibrin matrix of platelet-rich fibrin (PRF), from which they are slowly released into the surrounding tissue [16, 29, 14, 18]. Schar et al. [29] showed that PRF-associated growth factors, known as key coordinators of the physiological wound healing cascade, can enhance the wound cell-migration promoting healing. Raposio et al. [27] demonstrated already the efficacy of PRF augmentation on wound healing in chronic venous, diabetic and ischemic skin ulcerations compared to standard approaches. In a prospective study including 44 patients with persistent venous leg and diabetic foot ulcerations, Pinto et al. [26] emphasized the regenerative properties of PRF applied as monotherapy and the important role of patients’ own leucocytes. In addition, Vaheb et al. [34] demonstrated in a randomized, placebo-controlled, triple-blind study that PRF augmentation of split-thickness skin grafting in burns and chronic wounds significantly improved healing rates.

This pilot study reports for the first time the use of PRF for the treatment for dSSI after spinal instrumented surgery. Complete wound healing was achieved in all of our 12 consecutive patients between 14 and 21 days after the second revision using PRF. At a median follow-up of 8 months (range, 6–18 months), no recurrence of dSSI was observed and no complications were encountered in any case. In addition, instrumentation was preserved in all patients.

Readers might argue that the second surgical debridement alone combined with continuous pathogen-specific antibiotic treatment could explain the elimination of the dSSI and successful wound healing in our cases. However, large studies evaluating management strategies for dSSIs after spinal surgery with preservation of the instrumentation consistently reported the necessity of multiple surgical revisions, the use of antimicrobial moisture-retentive dressings and vacuum therapy as well as prolonged antibiotic treatment over months to achieve wound healing [1, 10, 12, 20, 21, 25]. Conversely, complete wound healing has observed within max 21 days in this cohort after the second surgical revision indicating a specific effect from PRF.

Limitations of this pilot study could be the small cohort and its retrospective design, which however could be understandable considering that in a 2-year period only 12 infected patients out of 24 needed an adjuvant PRF approach to achieve healing. The present study has not utilized an external matched control, which appears to be a common denominator with most of the published studies. The authors recognize the need for randomized controlled trials to demonstrate the efficacy of PRF to both treat and prevent dSSI after instrumented spinal surgery.

## Conclusions

This pilot single-center retrospective consecutive case series of patients with persistent deep SSI after instrumented spinal surgery suggests that PRF augmentation as an add-on therapy to standard debridement is safe and might provide a promising tool to achieve wound healing in critical cases. Using autologous biomaterial, PRF augmentation constitutes a simple and low-cost technique that can be easily applied in every operating room.
